# PPARγ ligands increase antileukemic activity of second- and third-generation tyrosine kinase inhibitors in chronic myeloid leukemia cells

**DOI:** 10.1038/bcj.2015.109

**Published:** 2016-01-08

**Authors:** E Glodkowska-Mrowka, A Manda-Handzlik, A Stelmaszczyk-Emmel, I Seferynska, T Stoklosa, J Przybylski, P Mrowka

**Affiliations:** 1 Department of Laboratory Diagnostics and Clinical Immunology of Developmental Age, Medical University of Warsaw, Warsaw, Poland; 2 Postgraduate School of Molecular Medicine, Medical University of Warsaw, Warsaw, Poland; 3 Department of Hematology, Institute of Hematology and Transfusion Medicine, Warsaw, Poland; 4 Department of Immunology, Medical University of Warsaw, Warsaw, Poland; 5 Department of Biophysics and Human Physiology, Medical University of Warsaw, Warsaw, Poland

BCR-ABL1 tyrosine kinase inhibitors (TKIs) have revolutionized the therapy of chronic myeloid leukemia (CML) and converted it into a truly chronic disease. However, there is still a significant group of patients who do not fully benefit from this success, as they fail to achieve remission, suffer from serious adverse effects of the therapy or undergo relapse or progression. Failure to complete eradication of CML cells with the current state-of-the-art treatment results from insensitivity of leukemia stem cells (LSCs) to TKIs.^1^ Knowing that more efficient inhibition of BCR-ABL1 with newer generations of TKIs is not able to cure the disease, a significant part of research effort has been redirected to find a way to effectively target LSCs. Therefore, many research groups have turned their interest into combination therapies, thereby allowing for interference with various signaling pathways.^2, 3^

Recent report by Prost *et al.*^4^ presented interesting data on erosion of LSCs pool by activation of peroxisome proliferator-activated receptor gamma (PPARγ), a transcription factor involved in the regulation of metabolism. It was shown that pioglitazone, a synthetic PPARγ ligand used in the treatment of diabetes, can stimulate proliferation of quiescent LSCs isolated from patients in chronic phase (CP) of CML. In this mechanism, the addition of pioglitazone to imatinib has induced complete and sustained molecular response in CML patients.

Independently, we performed a comprehensive analysis of the influence of PPARγ ligands on antileukemic properties of second- and third-generation TKIs in CML cells, which complement and extend data published by Prost *et al.* We have shown that addition of pioglitazone to TKIs (dasatinib, nilotinib and ponatinib) significantly decreased clonogenic potential of K-562 cells (Figure 1a, upper panel). The addition of pioglitazone affected not only the number but also the size and morphology of the colonies (Figure 1a, lower panel). Next, we investigated the efficacy of the combination of pioglitazone and ponatinib against CD34+ progenitor cells obtained from CML patients in CP (*n*=2) and blastic phase (BP; *n*=2) (Figure 1b). Colony formation was significantly inhibited by co-administration of pioglitazone and ponatinib when compared with the drugs alone. Similar increase in antileukemic efficacy of the studied TKIs was observed in cytotoxic assays in K-562 cells for four synthetic PPARγ agonists—thiazolidinediones (TZDs): pioglitazone, ciglitazone, troglitazone and rosiglitazone (Figure 1c).

Cytometric cell cycle analysis after propidium iodide staining revealed that 24-h incubation with pioglitazone and TKIs increased cell cycle arrest in G0/G1 from 66 to 73% for ponatinib, from 72 to 80% for nilotinib and from 71 to 86% for dasatinib (results calculated for cell cycle itself excluding subG1 phase). The addition of pioglitazone sensitized CML cells to TKIs as observed by increased number of K-562 cells in subG1 phase in TKI+pioglitazone group (Figure 2a). Cell cycle arrest was confirmed by western blotting analysis of p27 (Figure 2b). In consequence, pioglitazone significantly increased proapoptotic activity of TKIs as observed in increased cleavage of caspase 3 and PARP (western blotting, Figure 2b). To asses functional symptoms of induced cell death, a luminescent caspase 3/7 activity assay was performed on K-562 cells showing ~50% increase in caspase activity after addition of pioglitazone in comparison with TKIs alone (Figure 2c). Pioglitazone alone did not significantly affect cell cycle nor induced apoptosis (Figure 2).

Our results indicate that TZDs can not only eradicate quiescent LSCs as observed by Prost *et al.*^4^ but also increase apoptotic death of non-quiescent progenitors and differentiated CML cells, possibly facilitating the achievement of molecular response. Synergism between pioglitazone and second- and third-generation TKIs presented in our data suggests that the combination treatment can be successfully applied also in patients resistant to the first- or second-line therapy. Moreover, we have shown that the combination of pioglitazone and TKIs is a potent modality not only in CP but also in BP, including cells clinically resistant to the therapy (Figure 1b), which further confirms possible utility of PPARγ agonists in elimination of proliferating progenitors. It is especially interesting in the light of multiple clinical data suggesting that the rate of BCR-ABL1 decline as a result of TKI therapy may be important in achievement of major molecular response.^5^ Therefore, an increased potency of TKIs in combination with pioglitazone in eradication of BCR-ABL1-positive progenitors may give additional clinical advantage.

Considering pleiotropy of PPARγ and multiple off-target effects of TZDs, it is likely that their combination with TKIs will interfere with multiple signaling pathways. Prost *et al.*^4^ not only focused on STAT5 but also observed significant upregulation of OCT1 by pioglitazone, which could be responsible for increased intracellular concentration of imatinib. In these settings, OCT1 overexpression did not affect LSCs pool. Still, this mechanism might affect CML progenitor cell pool, similarly to our previous observations, showing that modulation of drug transporters activity by statins increases intracellular concentration of imatinib and potentiates its antileukemic efficacy^6^ that translates into higher rate of MR4.5 in patients on statin and imatinib.^7^

From clinical point of view, therapy with clinically available TZDs (pioglitazone or rosiglitazone) may raise some doubts. Rosiglitazone has been withdrawn from European market (although it is still available in the United States) because of reports on increased cardiovascular risk, whereas pioglitazone has been correlated with increased risk of bladder cancer. On the other hand, these potential adverse effects are still not unambiguously confirmed and were observed only after long-time treatment. The benefit of such treatment in patients with leukemia can overweight potential risk, and therefore the use of TZDs (including withdrawn troglitazone) can be justified. Moreover, pioglitazone is known to reduce cardiovascular risk in various clinical settings and is currently tested for secondary prevention after ischemic stroke in patients with diabetes.^8^ This protective effect might be beneficial in relation to the risk of serious cardiovascular side effects of TKIs.

Prost *et al.*^4^ showed that TZDs mainly influence LSCs. Our data add new information that this treatment modality might be also effective against progenitor cell pool (including advanced stages of CML) and not only in the context of imatinib treatment but also in combination with second- and third-generation TKIs. We believe that introduction of PPARγ agonists to the therapy may constitute a real breakthrough, finally leading to the cure of CML.

## Figures and Tables

**Figure 1:**
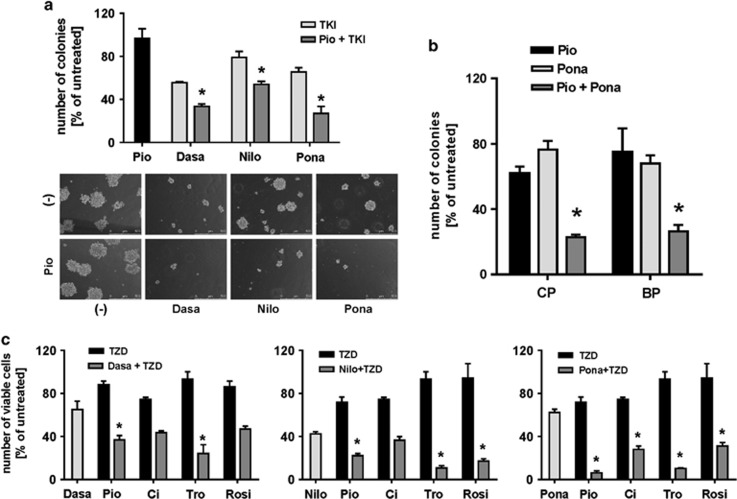
PPARγ agonists increase antileukemic activity of second- and third-generation TKIs. Pioglitazone increases antileukemic effects of TKIs (dasatinib, nilotinib and ponatinib) against K-562 CML cell line as observed in results (graph and images) of colony-forming assay (**a**). The effect of combination of pioglitazone and ponatinib was observed against primary CD34-positive cells isolated from patients in chronic (CML-CP) and blastic phase (CML-BP) (**b**). Both pioglitazone and other PPARγ ligands also exerted comparable effect against CML cells (K-562) when combined with TKIs as measured by cytotoxic assay after 48-h incubation with the drugs (**c**). *P<0.05 (analysis of variance and Tukey's *post hoc* test). Ci, ciglitazone (100 μM); Dasa, dasatinib (1 nM); Nilo, nilotinib (20 nM); Pio, pioglitazone (100 μM); Pona, ponatinib (1 nM); Rosi, rosiglitazone (100 μM); Tro, troglitazone (50 μM).

**Figure 2:**
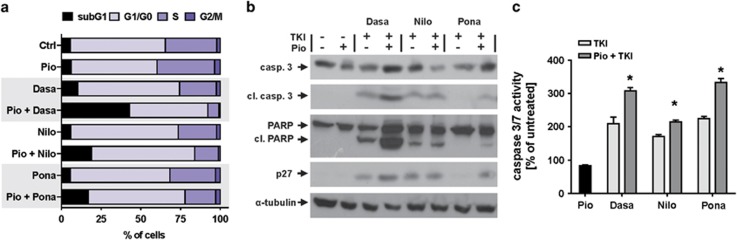
Pioglitazone increases TKI-mediated cell cycle arrest and apoptosis in CML cell line K-562. The addition of pioglitazone for 24 h induced cell cycle arrest in G0/G1 and sensitized K-562 cells to TKIs as observed by increased number of cells in subG1 phase in TKI+pioglitazone group (**a**). Cell cycle arrest was confirmed by increased expression of p27 (**b**). Pioglitazone significantly increased proapoptotic activity of TKIs as observed in western blotting (cleavage of caspase 3 and PARP) (**b**) and increased activity of caspase 3/7 in luminescent assay (**c**). *P<0.05 (analysis of variance and Tukey's *post hoc* test). Casp. 3, caspase 3; cl. casp. 3, cleaved caspase 3; cl. PARP, cleaved PARP; Dasa, dasatinib (1 nM); Nilo, nilotinib (20 nM); Pio, pioglitazone (100 μM); Pona, ponatinib (1 nM); TKI, tyrosine kinase inhibitor.
